# Qualitative study about the ways teachers react to feedback from resident evaluations

**DOI:** 10.1186/1472-6920-13-98

**Published:** 2013-07-16

**Authors:** Thea van Roermund, Marie-Louise Schreurs, Henk Mokkink, Ben Bottema, Albert Scherpbier, Chris van Weel

**Affiliations:** 1 Department Primary and Community Care, Radboud University Nijmegen Medical Centre, Nijmegen, Route number 166, Postbus 9101, Nijmegen 6500 HB, the Netherlands; 2 Institute for Medical Education, Faculty of Health, Medicine and Life Sciences, Maastricht University, Maastricht, the Netherlands; 3 Department Primary and Community Care, Radboud University Nijmegen Medical Centre, Post Graduate Training for Family Medicine, Nijmegen, the Netherlands; 4 Department Primary and Community Care, Radboud University Nijmegen Medical Centre, Post Graduate Training for Family Medicine, Nijmegen, the Netherlands; 5 Dean Faculty of Health, Medicine and Life Sciences, Maastricht University, Maastricht, the Netherlands; 6 Department Primary and Community Care, Radboud University Nijmegen Medical Centre, Nijmegen, the Netherlands; 7 Australian Primary Health Care Research Institute, Australian National University, Canberra, Australia

**Keywords:** Teachers, Professional development, Feedback, Role modeling

## Abstract

**Background:**

Currently, one of the main interventions that are widely expected to contribute to teachers’ professional development is confronting teachers with feedback from resident evaluations of their teaching performance. Receiving feedback, however, is a double edged sword. Teachers see themselves confronted with information about themselves and are, at the same time, expected to be role models in the way they respond to feedback. Knowledge about the teachers’ responses could be not only of benefit for their professional development, but also for supporting their role modeling. Therefore, research about professional development should include the way teachers respond to feedback.

**Method:**

We designed a qualitative study with semi-structured individual conversations about feedback reports, gained from resident evaluations. Two researchers carried out a systematic analysis using qualitative research software. The analysis focused on what happened in the conversations and structured the data in three main themes: conversation process, acceptance and coping strategies.

**Results:**

The result section describes the conversation patterns and atmosphere. Teachers accepted their results calmly, stating that, although they recognised some points of interest, they could not meet with every standard. Most used coping strategies were explaining the results from their personal beliefs about good teaching and attributing poor results to external factors and good results to themselves. However, some teachers admitted that they had poor results because of the fact that they were not “sharp enough” in their resident group, implying that they did not do their best.

**Conclusions:**

Our study not only confirms that the effects of feedback depend first and foremost on the recipient but also enlightens the meaning and role of acceptance and being a role model. We think that the results justify the conclusion that teachers who are responsible for the day release programmes in the three departments tend to respond to the evaluation results just like human beings do and, at the time of the conversation, are initially not aware of the fact that they are role models in the way they respond to feedback.

## Introduction

### Background

Currently, one of the main interventions that are widely expected to contribute to teachers’ professional development is confronting teachers with feedback from resident evaluations of their teaching performance. This expectation is based on the general educational theory that feedback fosters self-regulated continuous learning and performance improvement [1-5].

As most studies about feedback and the way feedback works concern students, little is known about teachers’ responses to feedback [6-10]. Knowledge about the teachers’ responses could be not only of benefit for professional development. Equally important is the fact that teachers are expected to act as role models and this includes role modeling in the way teachers respond to feedback. Therefore, research about professional development should include the way teachers respond to feedback.

### Theory and expectations

In this section, we first try to explain the relationship between feedback, self-regulation and the importance of role modeling within these two.

Feedback is defined by Van de Ridder et al. as “specific information about the comparison between a trainee’s observed performance and a standard, given with the intent to improve the trainee’s performance” [4]. In literature, one can find a variety of expectations about the ambiguous ways in which feedback influences self-regulation. Research about feedback emphasizes the importance of meeting with certain delivery conditions such as timing and report format. Although feedback intervention theories advocate textual feedback, various studies show that text formats do not necessarily result in increased improvement compared to numerical formats [11-13]. Numerical formats provide for a quick overview of results. Details and explanations follow when necessary [4,10,14]. In their reviews, Kluger and DeNisi [7,8] as well as L’Hommedieu et al. [15] indicate that expectations about the effects of feedback in general are often not realistic and the desired effects in terms of performance improvement not self-evident. In educational settings outside medical education, there are signs that teachers only act upon feedback when they feel themselves forced to do so [16].

Self-regulation presumes an open response towards the results on which feedback is based [7-9,12,15]. According to Sargeant et al., openness strongly depends on the acceptance of feedback [14]. Therefore, the authors plead for making room for and exploring emotions. Feedback means a risk of being confronted with information that can affect their professional focus or goal. Subsequently, individuals react with coping behaviour that varies between more problems (task) focused, avoidance (self-efficacy) focused or more emotion (self) focused [7,17-22]. Coping behaviour is defined as *“constantly changing cognitive and behavioural efforts to manage specific external and/or internal demands that are appraised as taxing or exceeding the resources of the person”*(p. 141) [22]. Problem focused coping include strategies that lead to directly resolve challenges, for instance participating in professional development activities. Avoidance focused coping refers to strategies that prevents one from feeling not competent. Emotion focused coping include strategies that lead to self-esteem and a positive self-image. According to Parker [19,20] and Runhaar et al. [23], the higher teachers’ self-esteem and self-efficacy, the more open they respond to professional activities that aim at problem solving and learning and that includes receiving feedback. Teachers want to succeed in their job, just like everyone else, and the two main goals they strive to attain appear to be “mastery” (i.e. I want to succeed) and “failure avoidance” (i.e. I don’t want to fail) [5,24,25]. Self-regulation depends also on the focus of the coping strategies and the main goal. Subsequently, being a role model in the way that teachers respond to feedback is not self-evident.

Role modeling has been the most important way to initiate young doctors into the profession since centuries. Nowadays, the high professional demands of self-regulation seem to enhance the residents’ need for strong positive role models in their learning environments Moreover, being a good role model is viewed as an essential part of professionalism [26-30]. In this view, role modeling includes being someone who shows and shares what it is like to be a doctor or a teacher [26,31]. The teachers’ attitudes and responses to feedback, mostly gained from resident evaluation, not only influence themselves but also whether residents perceive their teachers as role models, i.e. as good doctors as well as good teachers and persons [32,33].

In this study, we explore the responses of teachers to feedback reports based on resident ratings, in order to derive helpful suggestions for supporting self-regulated learning and to learn about the way teachers act as role models while receiving feedback. The study reported in this paper is part of a larger project, in which we developed an evaluation instrument for teachers in postgraduate day release programmes for general practice residents. For the full duration of their training, residents come to the academic department to attend the day release programme one day every week, including different types of small group sessions: reflection on the residents’ practice experiences and sessions devoted to medical topics and topics that are approached from a psychological perspective, such as doctor-patient communication. The programme also comprises individual appraisal interviews. During the three years of their training, groups of ten to twelve trainees work with the same teachers, usually a general practitioner (GP) and a behavioural scientist (BS), who act as teachers (curriculum topics) as well as coaches in the different types of sessions (reflection sessions in groups and individual appraisal interviews).

In a qualitative study, we address the following research questions:

–   How do teachers respond to the feedback reports immediately after reading them?

–   Are they (or do they act as) role models in the conversation processes?

## Method

### Design

We designed a qualitative study with semi-structured individual conversations, aimed at exploring teachers’ reactions to feedback reports based on resident ratings of their performance [34].

### Ethical approval

This study has been carried out in the Netherlands in accordance with the applicable rules concerning the review of research ethics committees and informed consent (The Research Ethics Committee of the Radboud University Nijmegen Medical Centre, Filenumber CMO 2012/412).

### Population

The study was conducted among teachers of the day release training programme for general practice residents.

### Data collection

In a Delphi study with residents and teachers, we developed an evaluation instrument for each of the different components of the programme: reflection on experiences, medical topics and progress interviews. The items were rated on a ten point scale, following the Dutch grading system. The residents completed the evaluation forms on several occasions.

When the evaluation questionnaires were being developed, the teachers indicated that they would like to be informed not only about their own but also about their colleagues’ performances, and therefore the evaluation reports for individual teachers contained not only their own average item scores but also those of the other participating teachers. We invited teachers from the three participating departments to discuss their individual feedback reports in a one-to-one meeting with one of the researchers (TR) in their own department. A pilot study we conducted showed that, before interpreting the results, the teachers needed some explanation about the structure of the report. Table 1 shows a fragment of a report.

**Table 1:** Report fragment

**Results evaluation of the reflection sessions**		
Date:		
Item/ standard	Indiv	Group
How enthusiastic was your teacher?	8.3 *	7.9
	7 - 10 **	5 - 10
Did your teacher show sensitivity towards the atmosphere in the group?	8.3	8.0
	7 - 10	4 - 10
Did your teacher show leadership?	8.7	7.8
	7 - 10	2 - 10
Did your teacher stimulate scientific reflection?	7.3	7.0
	2 - 9	1 - 10

The report was not given to the teachers until the meeting with the researcher, who gave the necessary explanations at the start of the conversations. The meetings lasted twenty to sixty minutes, depending on the need of the participants. The interview format consisted of three main topics in order to monitor the information: recognition of the results, reactions to the results, intentions with the results.

Because participants could be jumping into socially desirable answers in their responses to the feedback, the researcher used open questions and did not interrupt the respondents. When things were not clear, clarifying questions were asked (f.i. “what do you mean by….”or “I heard you say …. can you explain that”. In the information that was provided at the start of the evaluation study and every interview, the researcher indicated that participating in the evaluation study and the interviews would have no consequences, that the results would be presented anonymously. Furthermore, at the beginning of every interview, the teachers were individually asked for informed consent after reading the summary of their interview.

The conversations were tape recorded or, if a teacher objected, written notes were taken. As a respondent validation procedure, we asked the participants to comment on a summary of their interviews within one week after the interview and give consent for the use of the anonymised data for research and scientific publications [35]. Of 53 teachers participating, 50 agreed with the summaries and gave informed consent for using these for research activities and publication. Three participants declined to participate after the interviews. Two of them declined to participate because they did not agree with the evaluation results (mean results < 6) and one teacher (mean results >7) because she/he sympathized with these colleagues. From the 50 teachers that gave permission to use the interviews, two teachers suggested a minor change (they added a short explanation).

### Data analysis

The interviews were audiotaped and transcribed verbatim and entered into qualitative data analysis software (AtlasTi). Two researchers (TR and MLS) developed an analysis method and discussed this with the other authors. After agreement, they started the narrative analysis independently, focusing on what was happening in the conversations and, after that, how teachers narrated their experiences with the feedback reports [36].

The “what happened” was analyzed by looking at the conversation process. TR and MLS compared and discussed our interpretations, alternating between reading and discussing each other’s notes and agreed that the two main themes were: atmosphere and patterns [37].

“How teachers narrated their experiences” was analyzed as follows. The same two researchers independently coded the summaries. They used discussion about the differences in codes to reduce and refine these into one code book [38]. After two research meetings, we determined the categories on the basis of discussion and, subsequently, the main themes: acceptance and coping strategies. In order to illustrate the findings, quotations that related to the themes were discussed. Table 2 presents an overview of categories and themes.

**Table 2:** Categories and themes

**Categories**	**Main themes**
patterns	conversation process
atmosphere	
recognition	acceptance
attitude towards resident evaluation	
self-efficacy and resigning oneself	
Emotion focused	coping strategies
Avoidance focused	
Problem/ task focused	

## Results

The result section first presents characteristics of the participants (see Table 3). After that, we outline the conversation process and the way the teachers responded to the feedback reports, exploring the themes more in detail. Finally, the question of role modeling is answered.

**Table 3:** Characteristics of participants

	
Men	21
Women	29
General Practitioner	29
Behavioural Scientist	21
Teaching experience ≤3 years	18
Teaching experience 4–10 years	20
Teaching experience ≥ 11 years	12
Average teaching experience	6,5

### Key findings

#### ***The conversation process: atmosphere impressions and patterns***

At the start of the conversation, some teachers were curious and expressed their curiosity before seeing the results. Other teachers sat down, waited for the explanation, observed the results and reported what they saw. The researcher did not specifically ask about emotions but some teachers expressed their feelings and expectations as they saw the reports. Sometimes, teachers silently observed the outcomes and gradually admitted that the results reflected how things went.

*“I have some vague memories about these sessions. There were no spectacular events, no drama I guess. When such a session just passes by, I could try and give it a swing but will that help? So I recognize these ratings. Looking at the results I think “I just let it happen” and I don’t think I was sharp enough while working with this group and my colleague.” (3221 BS,* ≥ 11 *years of experience, below average results)*

Some teachers, however, did not start by observing the results, but by expressing criticism. After expressing their criticism, teachers enumerated some restrictions that they considered to be important for the interpretation of their results. They emphasized that the results should be regarded in the light of these restrictions and their criticism. Then they indicated that they did not remember the evaluated sessions. After that, they looked at the results. This reaction was not related to the results, as the teachers had not seen the results at the start of the conversation, but for instance to concerns about the responsibility of residents to collect the evaluation forms or with the teachers’ attitude towards the research afterwards. During the conversation, one teacher continued to be critical. The other critical teachers turned eventually to a more positive attitude, due to positive results and/ or the fact that they could express their criticism freely.

“Beforehand, I would like to give you some critical considerations with respect to this research.First, the resident who was responsible for collecting the evaluation forms was not competent for this. Second, afterwards, I think I did not want to participate in the research. Actually, it has overwhelmed me. Furthermore you should know that I teach two groups and I know that these groups think differently about me as their teacher. So you should take this into consideration while interpreting the results.…Well, overall I recognize these good results and that is nice to see, although it surprises me that the two groups barely differ like they do in other evaluations. I did not expect that”. (12219 BS, 2 year’s experience, above average results)

#### ***The conversation process: acceptance***

Teachers reacted calm and thoughtful, even when they were critical or saw some undesired results. Teachers recognised the results, either through comparison with the outcomes of other evaluations or because of the fact that they felt self-confident with the standards. We found various factors that influence acceptance: attitude towards resident evaluation, outcomes of other evaluations, self-efficacy toward a certain competency or resigning oneself to the outcomes. Teachers seemed to have ambiguous attitudes towards resident evaluations. On the one hand, they wanted to know how residents thought about their teaching performance and reflect on this, but on the other hand they felt that evaluation can be influenced by various factors, f.i. meeting with resident’s preferences.

We found a global pattern that seemed to lead to acceptance: after listening to the explanation of the reports, teachers observed the results, indicated that they recognised these, used various coping strategies to deal with the results and, subsequently, felt confident with the idea that they could not adapt to every standard. This was especially expressed for the scientific reflection item: mean scores were low, but according to the teachers, the reflection sessions mostly included personal reflection. Moreover, they did not feel competent to teach science and were fine with that (see Table 4).

**Table 4:** Global pattern in the conversations

Attitude*:*	*“It makes me think of how residents see me. But I don’t think I can say I’m doing well as a teacher. No, these ratings represent the satisfaction of the residents. But sometimes I do something they don’t like and I still think I’m doing the right things.”(3225 BS, 4 year’s experience, below average results)*
Recognition:	*“I recognise these results. After every teaching period we evaluate and these evaluations have the same outcomes. When I started my work as a teacher, the reflection sessions were problematic. Now it is more balanced and relaxed. I think I can see that in this report. I also want to say that I appreciate the items about personal characteristics.”(2217 GP, 4 year’s experience, average results)*
Self-efficacy and resigning:	*“Regarding science teaching I can say I’m done. I notice that, after ten years of teaching, I become a bit pig-headed. At this time I say: I know my qualities and I am more practical than a scientist.”(32226 BS, 9 year’s experience, above average results)*

#### ***Teachers with positive versus teachers with poor results***

Teachers with average or good results were happy and were determined to keep these good results. Most of them showed a positive attitude towards resident evaluations and self-confidence about their teaching qualities. However, some of them did not react only positively, although they recognised good results. They doubted their positive scores and showed a sceptic attitude towards resident evaluations, because they were convinced of the fact that residents had intentions with giving teachers high scores. Furthermore, they indicated that they did not take every evaluation equally seriously and their willingness to think about improvement plans seemed to depend on the origin of the ratings (see Table 5).

**Table 5:** Reactions to positive results

	
Positive attitude towards resident ratings:	*I like this feedback because it is only about me. That is different from the evaluations that we usually get as group teachers. The overall results are positive and I expected this. (32212 BS, 4 year’s experience, average results)*
Self-confidence:	*I feel confident with the reflection sessions. So it would surprise me if I got bad results. These positive mean scores, well, these are certainly in correspondence with reality (1215 GP, 7 year’s experience, above average results)*
Meaning of ratings:	*Does a high score mean that I ‘m doing well? I think that depends on how the residents perceive and interpret a situation. I think high ratings don’t make any sense and I mistrust these. I wonder if that is a resident who wants something from me. (22121 GP, 11 year’s experience, above average results)*
Performance improvement:	*If someone expects me to take notice of the results and make plans, then I want to know who gave me high or low ratings and what intentions did they have. Because changing behaviour is difficult and what will be the basis of the change? If a good and critical resident gives me poor grades then I will consider a change. But I do not have to take every evaluation seriously.(22115GP, 4 year’s experience, average results)*

Teachers with poor results tended to discuss the items instead of the results and tried to relate the results to causes outside themselves. They attributed the outcomes to characteristics of their resident group or assumed that residents did not understand the teachers’ approach.

But they did not necessarily react in a negative way. Teachers sometimes admitted that the low mean scores reflected their attitude towards the group or the items. Hesitantly, they started to reflect on their attitude and suggested that some residents rated their teaching performance low in order to confront them with an inappropriate attitude (see Table 6).

**Table 6:** Reactions to poor results

	
Criticize the items:	*I was not involved in developing this questionnaires and I think these could contain other items (1227 BS, 2 year’s experience, below average results)*
Attribution:	*Well, these results could reflect that fact that residents feel dependent. They could be afraid that we will reward them with poor grades. (32119 GP, 3 year’s experience, below average results*
Assumption:	*I am a role model in the way I ask questions, because I want the residents to ask good questions. I assume that the residents do not notice my intentions, because what I see in these results does not in any way reflect what I think to be important*. *(12212 BS, *≥ 11 *year’s experience, below average results)*
Self-reflection:	*These results confront me with the knowledge that I did not do my very best fort his group. If I look inside and if I’m honest, I know. (3221 BS, *≥ 11 *year’s experience, below average results)*

#### ***Coping strategies***

From our analysis, we identified emotion focused coping as well as avoidance focused and problem focused behaviours. We found many variations of emotion focused strategies. While reading the reports some teachers expressed their emotions and expectations. There was curiosity and disappointment, astonishment and joyfulness about but also mistrust of high grades. The ubiquitous coping strategies were to seek explanations or memories for the results, defending their performance or changing the standards to personal beliefs (see Table 7).

**Table 7:** Emotion focused coping strategies

	
Curiosity and excitement:	*I’m curious about the results. I think it is exciting and at the same time I see this report as a present (12111 GP 2 year’s experience, above average results)*
Disappointment:	*I recognize these results, but at the same time I’m disappointed, because they are lower than I hoped for. That strikes me and I don’t see myself that way.(32114, GP 3 year’s experience, below average results)*
Astonishment:	*The results for the item “did the teacher prepare for the progress interview” surprise me. We did really prepare ourselves. Didn’t they notice? (32222 BS, 1 year experience, below average results)*
Joyfulness*:*	*I see some items about personal characteristics and I like that. Those are exactly the characteristics I want to emit and so I’m happy to see good results in these items (22120 GP, 7 year’s experience, above average results)*
Mistrust*:*	*I mistrust good grades, because they come from residents who want to please me. These residents also try to hide themselves in the reflection sessions. They always act like that.(22121GP, 11 year’s experience, above average results)*
Expressing expectations:	*Some results are disappointing, for instance “was your teacher interested”, because I am really interested in the residents (12123 GP 7 year’s experience, below average results)*
Expressing expectations:	*I did not expect to get such high grades. I think this has to do with my feeling of self-efficacy. I think I’m not doing well, but apparently that’s a mistake. (12111 GP 2 year’s experience, above average results)*
Seek an explanation:	*Some of the residents in my group were negative. I remember some troublesome moments. That, I think, was hard to deal with* (*32120 GP, 4 year’s experience, average results)*
Memories of the session:	*I took my notes to this conversation so I can recall events and situations (32212 BS, 4 year’s experience, average results)*

Avoidance focused coping strategies were: rejecting the standards, avoiding questions and putting the results aside. When teachers rejected the standards, it was mostly in response to the evaluation of scientific reflection during reflection sessions; however, some teachers adapted the scientific standards, but did not feel competent. Teachers put the results aside or even rejected the evaluation outcomes outright when they thought that demands were too high. When teachers were confronted with the question if they had any intentions with the results, they mostly avoided this question (see Table 8).

**Table 8:** Avoidance focused coping strategies

	
Reject the standard:	*If I‘m expected to teach science with all those statistics, so if someone expects me to have that kind of expert knowledge, then I feel I’m not competent. I just can’t do it! (12111 GP, 2 year’s experience, below average results).*
Putting aside the results:	*I think it is alright. I know there are residents who are thrifty with giving grades and some are more generous. I guess that has to do with their characters (32119 GP, 3 year’s experience, above average results)*
Reject the outcomes:	*Well, there were schedule problems and, yes, I wonder what is best evaluation moment? Furthermore you have to take into account that there was a certain evaluation - weariness. And, yes, some residents have too high demands for us, teachers (32114 GP, 3 year’s experience, below average results)*
Avoidance (f.i. Do you have any intentions or do you intend to make any plans on the basis of these results?):	*Did you consider an item about the need for science; I mean the need of the residents? Because that could influence my results on that specific item. (1219 GP, 2 year’s experience, above average results)*

Problem focused coping strategies were identified as increasing efforts and reframing the problem. For this last strategy, teachers pointed for instance to the underlying problem of the current approach of the various sessions. We only found some weak signs of increased efforts in order to receive better evaluation outcomes. Teachers emphasized that they had strengths and weaknesses and that they were satisfied about their overall results. Furthermore, they expressed the need for discussing about “good teaching” with their colleagues because the items as well as the conversation had made them reflect about how to teach (see Table 9).

**Table 9:** Problem focused coping strategies

	
Reframing the problem:	*My points of interest are about leadership and scientific reflection. But I miss a basis for teaching science and I also think that our ideas about the reflection sessions lack a fundamental perspective on science. (32221 BS 2 year’s experience average results)*
Increasing effort*:*	*I really do want to change things. Being enthusiastic and being a role model are the things I want to improve. These evaluations are snapshots but I think these reports are important (32123 GP* ≥ 11 *year’s experience above average results)*
Need for discussion:	*If I look at my results, I could only think “I’m doing well”, so that’s on a personal level. But this report also makes me think about “good teaching” and that is equally important. Something like: how can I be a role model as a behavioural scientist? I would really like to discuss this with my colleagues in the institute (2229 BS* ≥ 11 *year’s experience, above average results)*

### Role modelling

Looking at the conversation process, teachers observed the results, asked themselves questions and looked for answers by using various coping strategies. Data analysis showed that teachers tended to emotion and avoidance focused strategies. They accepted the results after reflection, stating that they cannot achieve every standard that is presented by the items. Some teachers indicated that they would like to see only high results, but they avoided the question of how to get there. Teachers tended to focus on the overall results (mean score 7–8) and seemed to be satisfied.

We found various strategies, but there seemed to be a hierarchy of strategies. Looking for explanations for the results and attribution (to external factors) appeared to be the most used strategies. Table 10 presents the top five of most used coping behaviours.

**Table 10:** Top five of coping strategies

	
1.	Seeking explanations
2.	Attribution to external factors
3.	Changing standards to personal beliefs
4.	Expressing emotions
5.	Defending performance

## Discussion

From resident evaluations, feedback conversations with individual teachers were conducted with the aim of exploring various aspects of teachers’ reactions to reports that present resident ratings of their individual and their colleagues’ performances, made after a period of specific sessions. The data were analysed in two ways to answer the research questions: a narrative analysis of the conversation processes (what happened) and a coding and structuring analysis of the coping strategies (how do teacher respond).

The start of the conversations differed between teachers. Teachers who had a critical attitude expressed their criticism before observing the results. Following the outcomes of the studies of Sargeant et al. [14] and Eva et al. [10], the researcher engaged the teachers as partners in the feedback conversation. She explored the criticism, asked teachers about their feelings when they allowed to do so and this helped teachers to eventually focus on the reports. The criticism concerned the research procedures and the fact that, afterwards, teachers felt obligated to participate, although the researcher emphasized during the information phase that participation was voluntary.

During the conversation, it seemed as if teachers were talking to themselves while using all kinds of coping strategies, trying to make space for interpretations and acceptance. In literature, this process is described from the perspective of symbolic interactionism by Mead [39], Blumer [40] and recently, more deeply, from the dialogical self – theory of Hermans [41,42]. Hermans points at the way people react to confrontations with themselves, what is going on inside them, behind the coping behaviours. In fact, receiving feedback is such a confrontation. Teachers reflected on the results and used all kinds of coping strategies to confirm not only the memories and expectations they had but also to confirm the image they have of their teaching and/or to feel competent. This could explain why we found merely emotion focused and avoidance focused strategies and not so much problem/task focused coping. The feedback and interpretations of the feedback appeared not (immediately) to lead to mastery or failure avoidance, as categorized by Seifert [25] and Butler and Winne [5]. Instead, we seem to have identified forms of inner speech that are explained in the dialogical self theory by Hermans [41,42] in the process of receiving, interpreting and accepting the feedback. Inner speech from the teacher himself (“Am I the teacher that is described” or “Do I want to do what is described in the items”), and from significant others who take a meaningful position inside a teacher (“Who said what about me as their teacher” or “Whose ratings will I take seriously”). Along these lines teachers seemed to engage their reports and tried to give answers to their own questions, aroused by the results.

This study confirms that acceptance is influenced by numerous factors: self-efficacy, attribution, reflection on one’s abilities and beliefs, and attitude towards improving performance in general [16,23,43-47]. For most of the teachers, the results were not surprising not only because they had been evaluated earlier but also because they did not expect to perform better at some points and therefore, had no intentions to increase their efforts. According to Bandura, the likelihood that people will act on the outcomes they expect prospective performances to produce depends on their beliefs about their earlier ability to produce those performances.

The study also shows that acceptance can appear in different natures and that this nature can lead to reflection but not necessarily to intentions for improvement. According to our findings, acceptance merely implied that teachers resigned themselves to the fact that they cannot know everything or be competent at every domain, instead of implying that teachers would increase their efforts. Questions about plans or intentions with the feedback results were mostly avoided. We conclude that making plans for improvement, amongst other things, depends on the nature of acceptance. However, teachers could decide to use the reports for improvement sometime after the conversations, but research on the ultimate effects of the feedback and the conversations was beyond our scope.

Finally, we turn to our second research question regarding role modeling. In our study, teachers reacted to feedback just like many others, f.i. students and residents. They interpreted the results in such a way that it felt comfortable, acceptable and supporting for their self-efficacy. In the introduction of this study, we stated that role modeling includes being someone who shows and shares what it is like to be a doctor or a teacher [26,31]. In their review, Jochemsen et al. [32] found that a positive role model is perceived as “someone who is admired for being, or acting as, a professional or as someone who inspires and teaches while carrying out other tasks”. In these perspectives, role modeling refers to a charismatic authority and this type of authority not only depends on “just being a doctor/teacher” but also on the acceptance of ‘one being a doctor/teacher” by (significant) others. This acceptance of significant others is, as we saw earlier in this discussion, a delicate problem that affects the self of the doctor/teacher.

By exploring the teachers’ reactions, we think that the conversations yielded useful insights into the variety of ways in which feedback can be received and dealt with by teachers of the day release training programmes. These insights can be used to inform faculty development initiatives as well as stimulate the reflection about role modeling for this group of teachers.

### Strengths and weaknesses

As far as we know, this study is the first to examine the reactions of teachers to resident feedback within the setting of the GP day release training programmes. The first researcher conducted the conversations and participated in the analysis as well. We tried to minimise possible interpretation effects by extensive involvement of a second researcher in the analysis and of the co-authors in the subsequent phases. The study afforded a unique opportunity to witness how teachers respond to evaluation reports and reflect on their teaching behaviour by taking time to discuss feedback in an open atmosphere.

## Conclusion

We think that the results justify the conclusion that teachers who are responsible for the day release programmes in the three departments tend to respond to the evaluation results just like human beings do. The next, most obvious, conclusion could be that teachers seemed to be unable to take their role as good feedback receivers and continuous learners or forgot to do so in the stress that comes along with receiving feedback. But with a closer look and an open mind, we could equally conclude that they are really good role models, because even professionals are human beings and this is a natural way of responding to feedback. However, being a good role model means that, at some point, teachers will have to take their role as good feedback receivers and show commitment to it and have a positive critical attitude towards learning, i.e. professional development.

Our study not only confirms that the effects of feedback depend first and foremost on the recipient but also enlightens the meaning and role of acceptance.

Further research might investigate processes that tackle teachers’ professional development from a motivational perspective. Another important area for further research would be evaluation methods that can lead to alignment of expectations and aims with residents and compare these with one-way feedback processes like the evaluation that was the subject of the present study.

## Competing interests

The authors declare that they have no competing interests.

## Authors’ contributions

TR was principal researcher and wrote the article. MLS contributed to the data analysis. BB and HM were co-author. AS and CW were co authors and supervised the study. All authors read and approved the final manuscript.

## Pre-publication history

The pre-publication history for this paper can be accessed here:

http://www.biomedcentral.com/1472-6920/13/98/prepub
